# Diagnostic performance of verbal fluency measures: a cross-sectional study in the stages of cognitive continuum

**DOI:** 10.1186/s13195-026-01963-3

**Published:** 2026-01-20

**Authors:** Mihály Unoka, Dalida Borbála Berente-Kerestély, Melinda Becske, Andras Attila Horvath

**Affiliations:** 1 Faculty of Medicine, Semmelweis University, Üllői út 26, Budapest, 1085 Hungary; 2 Nyírő Gyula National Institute of Psychiatry, and Addictology, Neurocognitive Research Centre, Budapest, Hungary; 3 Department of Anatomy Histology and Embryology, Semmelweis University, Budapest, Hungary; 4 HUN-REN, Research Centre for Natural Sciences, Budapest, Hungary; 5 Faculty of Medicine, University of Lisbon, Lisbon, Portugal; 6 Department of Neurosurgery and Neurointervention, Semmelweis University, Budapest, Hungary

**Keywords:** Verbal fluency, Semantic fluency, Verbal fluency discrepancy, Subjective cognitive decline, Semantic memory, Minor neuropsychological deficits

## Abstract

**Background:**

Verbal fluency (VF) measures are sensitive markers of advanced cognitive decline; however, the utility of the discrepancy score remains underexplored in the early stages of cognitive decline, such as subjective cognitive decline (SCD). This study evaluated semantic fluency (SF) and phonemic fluency (PF), as well as discrepancy score sensitivity, in clinical populations with SCD, mild cognitive impairment (MCI), and dementia.

**Methods:**

In this cross-sectional study, 193 older adults (72 healthy controls, 67 with SCD, 16 with MCI, and 38 with dementia) were consecutively recruited from the Nyírő Gyula National Institute of Psychiatry and Addictology, Hungary. Each participant underwent a comprehensive neurological interview and neuropsychological assessments. Semantic and phonemic fluency, along with their discrepancy, served as the primary outcome measures, defined as the number of correct words generated in one minute for each fluency type and the difference between them. Group differences were assessed using one-way ANCOVAs that controlled for age and education, and diagnostic classification performance was evaluated using a multinomial logistic regression model-based metric.

**Results:**

Relative to healthy controls, SCD showed significantly lower SF scores (β = − 2.267, *p* = .009) but no difference in PF (*p* = .493). Both fluency types were reduced in MCI and dementia, with semantic declines being especially pronounced. Multinomial logistic regression model identified dementia most accurately using SF (balanced accuracy = 0.81) and SCD using the discrepancy score (balanced accuracy = 0.61), while MCI classification was poor, likely due to a small sample size. These results underscore the potential of SF and its discrepancy with PF for early detection and differentiation of cognitive decline.

**Conclusions:**

This study demonstrates that individuals with SCD have deficits in semantic—but not phonemic—fluency, and that VF discrepancy scores detect SCD more effectively than semantic fluency alone. However, accuracy is only moderate and insufficient for use as a standalone clinical test.

## Introduction

Cognitive decline typically progresses from subjective cognitive decline (SCD) to mild cognitive impairment (MCI) to dementia [1, 2]. As most individuals with cognitive decline are in predementia stages [3], where prevention, early intervention or therapy is most effective [4, 5], early detection is essential.

Standardized neuropsychological batteries designed to detect MCI or dementia typically lack the sensitivity to capture the subtle deficits in SCD. However, some individuals with SCD may display subtle deficits on specific cognitive measures that do not meet MCI criteria [6] but still predict an increased risk of future cognitive decline [7]. Identifying these deficits and the most sensitive tests is crucial. We address this gap by evaluating verbal fluency (VF)—semantic (SF), phonemic (PF)—and their discrepancy as potential sensitive markers in SCD.

Semantic and phonemic VF measure distinct cognitive processes [8]. SF is more associated with semantic memory, while both fluency test types reflect executive function capacity [9]. In normal aging, there is an advantage in semantic tasks over phonemic tasks [10], which appears to reverse in MCI and dementia along the trajectory of AD. This discrepancy can be measured by the semantic-phonemic discrepancy score (subtracting the phonemic from the semantic fluency raw score). The discrepancy score controls for the standard components of the two fluency scores, primarily measuring semantic memory [11]. Semantic memory is one of the earliest cognitive functions affected in dementia [11], which raises the question: Can individuals with SCD be distinguished from healthy controls based on their raw semantic fluency scores or discrepancy scores?

In the current clinical study, we compared semantic and phonemic VF test scores across four groups: healthy, SCD, MCI, and dementia. We hypothesized that there would be a significant difference in SF between individuals with early cognitive decline and those with healthy performance, but not in PF. We also tested the ability of semantic, phonemic, and their difference (i.e., the VF discrepancy score) to differentiate between SCD individuals and other groups included in the study. We hypothesized that the discrepancy scores would offer the best discriminatory power.

In summary, our primary goals are (I) to compare semantic and phonemic fluency across healthy, SCD, MCI, and dementia groups, and (II) to determine if the discrepancy score enhances the discriminatory power for early cognitive decline, particularly in SCD.

## Methods

### Study population

The study included data from 205 participants of the AlzEpi Cohort Observational Library (ACOL) database at the Nyírő Gyula National Institute of Psychiatry and Addictology. The database comprises cognitively healthy elderly individuals, participants with SCD, individuals with MCI, and dementia. The database contains demographic, neuropsychological, neurophysiological, neuroimaging, cerebrospinal fluid, clinical, and biometric data and is part of the Euro-Fingers database (http://www.eufingers.com).

To control for factors other than MCI and dementia that negatively affect cognition, further exclusion criteria included hypothyroidism, untreated vitamin B12 deficiency, major systemic illness, liver disease, renal failure, schizophrenia, major depression, electroconvulsive therapy, psychoactive drug use, substance or alcohol abuse, prior central nervous system infection, HIV infection, syphilis, loss of consciousness due to head trauma, hydrocephalus, severe cerebrovascular disease (e.g. white matter disease, cortical ischemia), and demyelinating conditions. Because of missing relevant demographic data, we excluded 13 participants from the analyses after careful consideration.

We therefore completed statistical analyses using data from 193 participants: 72 healthy controls, 67 individuals with SCD, 16 with MCI, and 38 with dementia. Every participant was a native Hungarian. All participants gave their written informed consent. The Hungarian Medical Research Council authorized our research (reference number: V/5831 3/2021/EKU). A comprehensive dementia screening protocol, encompassing a medical history review, psychiatric and neurological evaluations, blood tests, extensive neuropsychological testing, brain structural MRI, and resting-state functional MRI, was administered to every participant. MCI and dementia patients also went through CSF analysis. Based on the results, a multidisciplinary medical team made the diagnosis. Healthy control individuals reported no memory complaints, exhibited a negative neurological status, and scored within the normal range on neuropsychological tests. Their MRI and blood tests were also normal. We defined SCD according to the SCD-I consensus [12, 13]: self-experienced, persistent decline in cognitive capacity compared with a previously normal level, unrelated to an acute event; and normal age-, sex-, and education-adjusted performance on standardized cognitive tests that are used to classify MCI, dementia (RAVLT and MMSE in this case). Individuals were excluded if they met criteria for MCI, or dementia, or if symptoms were better explained by a psychiatric or neurological disorder, medical condition, medication, or substance use. SCD-plus is SCD characterized by features that indicate a higher prior probability of preclinical Alzheimer’s disease. All SCD participants attended our memory clinic seeking medical help and reported primarily memory-related subjective decline; therefore, each participant fulfilled ≥ 2 SCD-plus features (help-seeking; memory-domain complaint) [13]. MCI patients were diagnosed using the Petersen criteria. Subjective memory deficits in these participants were objectively confirmed by cognitive tests [14]. However, the level of cognitive impairment did not affect their independent daily living [14]. Overall, individuals with significant vascular lesions on the MRI (Fazekas score > 1) were excluded from the current analysis. Brain atrophy was confirmed by MRI in the MCI and dementia groups (Scheltens score > 0; Koedam score > 0, Global atrophy score > 0).

### Neuropsychological examination

Trained professionals in neuropsychology administered the neuropsychological tests. Global cognitive performance and major cognitive subdomains (orientation, attention, memory, verbal fluency, language, and visuospatial abilities) were assessed using the validated Hungarian version of the Addenbrooke Cognitive Examination (ACE) [15]. ACE scores were used to describe cognitive profiles and were not used for diagnostic classification, apart from the derived MMSE total. The orientation subdomain assesses both spatial and temporal orientation. During the attention task, participants must, for example, count backward from 100 in steps of seven, and the memory part assesses both anterograde and retrograde memory functions. The verbal fluency part of the ACE evaluates both phonemic and semantic fluency. For the phonemic fluency task, participants were asked to list as many words beginning with the letter ‘M’ as they could in one minute. For the semantic fluency task, the participants had to list as many animals as they could in 60 s. Further, our statistical analysis used the number of words listed by participants (raw fluency scores) for each verbal fluency task. The verbal fluency discrepancy score was derived by subtracting the raw phonemic fluency score from the raw semantic fluency score. The language part assesses, for instance, understanding of commands, recognition of objects, and sentence formation. In contrast, the visuospatial subdomain assesses visual construction in the form of copying a cube and two overlapping pentagons. The test is finished with a clock drawing test.

The Mini-Mental State Examination (MMSE) was a part of the ACE test battery. It has a maximum score of 30 points [16]. It was employed as the primary neuropsychological tool to support dementia diagnosis, due to its widespread use in dementia research. Although many investigations have adopted a cutoff score of 26 to identify clinically significant dementia, our approach utilized an established adjustment that accounts for both educational attainment and age [17] (for the cut-offs, see Table 1 in this study [18]).

**Table 1:** *Demographic, clinical and verbal fluency characteristics of participant groups*

Independent Variables	Controls Mean (SD)	SCD Mean (SD)	MCI Mean (SD)	Dementia Mean (SD)	*p*-value
N	72	67	16	38	
Age	67.42 (8.02)	65.57 (9.71)	74.63 (7.86)	77.00 (6.66)	<.001
Education (Low/Moderate)	18	19	6	20	.020
Education (High)	54	48	10	18	
Gender (Female)	46	48	7	24	.343
Gender (Male)	26	19	9	14	
ACE	93.83 (3.65)	92.43 (4.81)	81.88 (6.85)	62.53 (11.96)	<.001
MMSE	28.63 (1.03)	28.60 (1.17)	27.44 (0.63)	21.26 (3.95)	<.001
STAIS	36.31 (10.58)	39.62 (10.76)	42.00 (8.58)	43.71 (9.58)	.017
STAIT	40.29 (7.29)	46.35 (10.72)	42.00 (5.89)	43.52 (6.68)	<.001
BDI	3.81 (3.03)	7.14 (4.71)	5.81 (3.75)	8.04 (6.02)	<.001
SF (words/min)	23.04 (4.73)	21.07 (5.52)	14.06 (6.74)	9.79 (5.26)	<.001
PF (words/min)	14.88 (4.65)	14.39 (5.12)	10.88 (4.53)	8.03 (4.08)	<.001
VF discrepancy (SF − PF)	8.17 (5.09)	6.69 (4.43)	3.19 (5.65)	1.76 (4.25)	<.001

*Low education* Completed only elementary/primary school or dropped out early, *Moderate education* Completed secondary education (high school or equivalent), *High education* Attained post-secondary education (some college, bachelor's, or higher), *ACE* Addenbrooke’s Cognitive Examination, *MMSE* Mini-Mental State Examination, *STAIS* State-Trait Anxiety Inventory-State, *STAIT* State-Trait Anxiety Inventory-Trait, *BDI* Beck Depression Inventory, *SF* Semantic Fluency, *PF* Phonemic Fluency, *VF* Verbal Fluency

To objectively evaluate memory complaints consistent with the Petersen criteria, the validated Hungarian version of the Rey Auditory Verbal Learning Test (RAVLT) [19] was administered. Prior studies indicate that the RAVLT is highly sensitive for detecting a-MCI [20] because of the early involvement of verbal learning-based memory processes. In this test, participants first learn a set of 15 words (List A) that is repeated five times (RAVLT sum-5 reflects the total number of correct words over these five trials). They then receive a different set of 15 words (List B) for a single trial, followed by a recall test. Next, they are asked to recall List A again without repetition, and 30 min later, they attempt to recall it once more (RAVLT 7 quantifies the total correct words in this delayed recall) (for the cut-offs, see Table 1 in this study: [18]). RAVLT performance served as the objective diagnostic criterion for MCI.

To control for the potential hindering effect of depression and anxiety on cognitive function, we applied the Hungarian version of the 13-question Beck Depression Inventory (BDI) [21] and Spielberger State-Trait Anxiety Inventory (STAI) [22]. General daily anxiety was assessed using the Trait test of the State-Trait Anxiety Inventory (STAI-T). In contrast, the State test of the STAI (STAI-S) was applied to monitor anxiety levels during the neuropsychological evaluation.

Multidisciplinary panel assigned diagnostic categories using clinical interview, structured functional assessment, brain MRI, CSF where available, MMSE total derived from the ACE-embedded MMSE items, and RAVLT performance. All ACE total and subdomain scores — including semantic and phonemic fluency — were withheld from the adjudication panel and did not contribute to diagnostic assignment; only the derived MMSE total was provided. We used ACE subdomains solely to describe cognition and as outcome variables in analyses. Dementia was diagnosed clinically with functional impairment and supportive MMSE and MRI findings [16]; MCI followed Petersen criteria with objective memory impairment on the RAVLT and preserved activities of daily living and supportive MRI findings; SCD followed Jessen criteria (subjective cognitive decline with normal MMSE and RAVLT; SCD-plus features where present).

### Statistical analysis

All our statistical analyses were run in R Studio 4.4.2. We first performed descriptive analyses (means, standard deviations, and frequencies) to characterize the four diagnostic groups (healthy controls, SCD, MCI, and dementia). We examined group differences in demographic and clinical variables using chi-square tests, Fisher’s exact tests, and Kruskal–Wallis tests, as appropriate.

Next, we conducted one-way ANCOVAs to evaluate differences in semantic and phonemic fluency across groups while controlling for age and education. Significant main effects were further examined via post hoc pairwise comparisons and effect size estimates (partial eta squared).

To assess the predictive utility of each fluency measure (including the discrepancy score), we used multinomial logistic regression, estimating relative risk ratios (RRRs) while adjusting for age and education. Model performance was evaluated via confusion matrices and multi-class classification metrics (balanced accuracy, F1, recall, and specificity) in a one-vs-all approach. Finally, we constructed Precision–Recall curves (PRC) using a one-vs-all approach and calculated the area under the curve (AUC–PR) values for each diagnostic category to address class imbalances and understand the sensitivity of each measure to subtle cognitive decline.

Power analysis showed that, although the omnibus ANCOVAs and most post-hoc contrasts were strongly powered for MCI (0.70–1.00) and dementia (≈1.00), their ability to detect the subtler SCD effect was only good for semantic (0.76) and low for phonemic (0.11). The multinomial-logistic model echoed this pattern, yielding low power for the SCD VF-discrepancy coefficient (0.57) but adequate power for the corresponding MCI (0.84) and dementia (0.99) coefficients.

## Results

### Descriptive statistics

The study initially included 205 participants: 72 healthy controls, 67 individuals with subjective cognitive decline (SCD), 18 with mild cognitive impairment (MCI), and 48 with dementia. After excluding 13 participants lacking education data (critical for covariate adjustment), the final sample comprised 193 participants (72 controls, 67 SCD, 16 MCI, and 38 dementia). Demographic characteristics and clinical scores for the participant groups are summarized in Table 1. Significant group differences were found in age, education, and cognitive measures, including ACE and MMSE, as well as psychiatric measures such as STAI and BDI.

A Chi-Square test revealed no significant association between gender and group membership, χ2 = 3.34, p = 0.343. However, Fisher’s Exact Test indicated a significant association between education level and group (p < 0.001), with the healthy control and SCD groups having a higher proportion of participants with high education compared to the MCI and dementia groups. The Kruskal–Wallis test also showed a significant difference in age across groups, χ2 (3) = 62.58, p < 0.01, with the MCI and dementia groups being older on average than the healthy control and SCD groups. Given that group status showed significant associations with both education and.

Age was included as a covariate in the subsequent analyses. The details of the recruited individuals are summarized in Table 1.

The VLOM ratio from the ACE test effectively assessed overall cognitive impairment, distinguishing typical Alzheimer’s disease patterns from atypical or alternative dementia etiologies. Among the 38 participants with dementia, 4 (10.5%) had a VLOM ratio < 2.2 (suggesting atypical Alzheimer’s or alternative etiologies), 3 (7.9%) scored between 2.2 and 3.2, and the remaining 31 (81.6%) had VLOM ratios > 3.2, consistent with a typical Alzheimer’s pattern. In those 31 patients, CSF analysis supported Alzheimer's dementia pattern with positive amyloid and tau pathology, while in the remaining seven subjects, only one patient showed AD pathology. In the MCI group, CSF analysis confirmed MCI due to AD pathology in 9 cases.

After presenting the descriptive statistics, we first used ANCOVA models (controlling for age and education) to confirm whether semantic and phonemic fluency scores differed significantly across the four diagnostic groups. Building on these observed group differences, we subsequently performed a multinomial logistic regression to assess the predictive (i.e., diagnostic) utility of the fluency measures, including the VF discrepancy score, in classifying individuals into each diagnostic category. This sequential approach illustrates how initial group-level differences can translate into meaningful classification capabilities in a clinical context.

### Group comparisons via ANCOVA

All assumptions for ANCOVA were met, including homogeneity of regression slopes, linearity, homogeneity of variance, with normality confirmed and with no multicollinearity.

An ANCOVA was conducted to assess the effect of group membership (healthy controls, SCD, MCI, and dementia) on semantic and phonemic fluency scores. Age and education were included as covariates to control for potential confounding.

The analysis revealed a significant main effect of group on both outcomes — semantic and phonemic fluency scores (see Table 2). The effect size was large for semantic fluency and large but smaller for phonemic fluency (η^2^_partial = 0.01, 0.06, and 0.14 considered small, medium, and large effects).

**Table 2:** Analysis of Covariance (ANCOVA) for Semantic and Phonemic Fluency Scores

	Semantic Fluency				Phonemic Fluency			
	F	df	p	η^2^_partial	F	df	p	η^2^_partial
Group	34.56	3, 187	<.001	.36	10.92	3, 187	<.001	.15
Age	19.75	1, 187	<.001	.10	2.98	1, 187	.086	.02
Education	4.54	1, 187	<.01	.02	10.57	1, 187	<.001	.05

ANCOVA results for semantic and phonemic fluency scores. p-values are reported to three decimal places unless p <.001. η^2^_partial represents partial eta squared effect sizes. For context, the overall model test (model-level F and Multiple R2) fit well: semantic overall model test F(5,187) = 45.4, p <.001, R2 (multiple) = 0.55; phonemic overall model test F(5,187) = 16.1, p <.001, R2 (multiple) = 0.30

Both age and education significantly influenced semantic fluency, but with different effect sizes: age had a medium effect, while education had a negligible effect. On the other hand, age did not have a significant impact on phonemic fluency, though it trended toward significance. Education was found to have a substantial effect, with a small effect size, on phonemic fluency. Results are summarized in Table 2.

### Post hoc comparisons

**Fig. 1:**
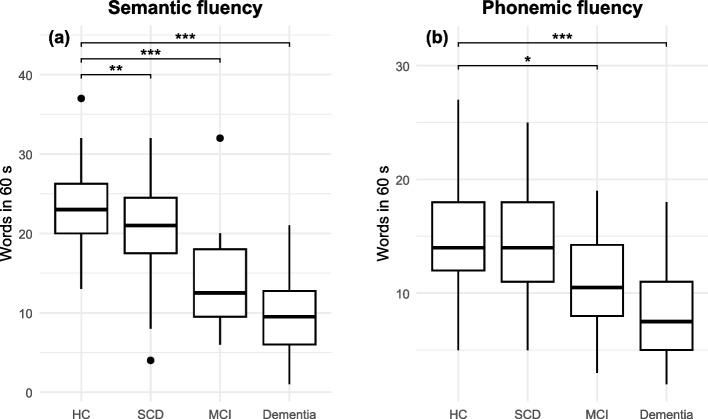
Boxplots illustrating semantic (**a**) and phonemic (**b**) fluency across diagnostic groups. Boxes show the inter-quartile range; horizontal lines mark the median; whiskers extend to 1.5 × IQR; points are outliers. HC = healthy controls (*n* = 72); SCD = subjective cognitive decline (*n* = 67); MCI = mild cognitive impairment (*n* = 16); Dementia (*n* = 38). Fluency scores represent the number of correct words generated in 60 s. Stars denote pair-wise contrasts from an ANCOVA that controlled for age and education: * *p* <.05, ** *p* <.01, *** *p* <.001. Axes are scaled independently for clarity

After adjusting for age and education, post hoc comparisons (Table 3) revealed that SCD participants scored significantly lower on semantic fluency than healthy controls, whereas phonemic fluency did not differ significantly. Specifically, the SCD group had substantially lower semantic fluency scores compared to healthy controls, whereas no significant differences emerged in phonemic fluency. In contrast, participants with mild cognitive impairment (MCI) and dementia exhibited robust deficits in both semantic and phonemic fluency. Additionally, increasing age was associated with a reduction in semantic fluency. At the same time, education had a positive influence on performance on both tasks, although its effect was more pronounced for phonemic fluency than for semantic fluency. Overall, these findings highlight that SCD is associated with an early decline in semantic fluency (Fig. 1). In contrast, phonemic fluency remains comparable to that of healthy controls, underscoring the sensitivity of semantic tasks in detecting subtle cognitive changes (Fig. 1).

**Table 3:** Post Hoc linear model results for verbal fluency performance in cognitive decline

Comparison	Dependent Variable	β	SE	t-value	p-value
SCD vs. Healthy Controls	Semantic Fluency	−2.267	0.854	−2.653	.009
MCI vs. Healthy Controls	Semantic Fluency	−7.381	1.422	−5.190	<.001
Dementia vs. Healthy Controls	Semantic Fluency	−10.942	1.107	−9.880	<.001
Age effect	Semantic Fluency	−0.193	0.043	−4.445	<.001
Education effect	Semantic Fluency	1.681	0.789	2.131	.0344
SCD vs. Healthy Controls	Phonemic Fluency	−0.535	0.779	−0.686	.493
MCI vs. Healthy Controls	Phonemic Fluency	−3.215	1.297	−2.478	.0141
Dementia vs. Healthy Controls	Phonemic Fluency	−5.548	1.010	−5.492	<.001
Education effect	Phonemic Fluency	2.339	0.719	3.252	.001
Age effect	Phonemic Fluency	−0.0683	0.0395	−1.728	.0857

All analyses controlled for age and education. P-values are reported to three decimal places unless *p* <.001

*SCD* Subjective Cognitive Decline, *MCI* Mild Cognitive Impairment, *SE* Standard Error

### Diagnostic classification analyses: multinomial logistic regression model

Given the significant differences revealed by our ANCOVA analyses, we next conducted a multinomial logistic regression to determine the extent to which the verbal fluency measures, including the VF discrepancy score, predict or classify individuals across the four diagnostic groups. In doing so, we aimed to move beyond merely identifying group differences to evaluating how effectively these measures function as diagnostic indicators of SCD, MCI, and dementia relative to healthy controls, while adjusting for age and education.

**Fig. 2:**
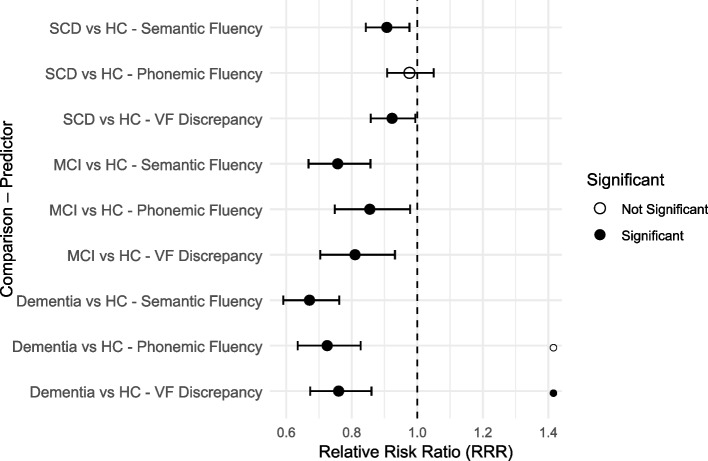
Relative risk ratios for verbal‑fluency measures predicting diagnostic status. Forest plot showing the relative risk ratios (RRR) and 95% confidence intervals for each verbal fluency measure (semantic, phonemic, discrepancy) predicting group membership (SCD, MCI, dementia) relative to healthy controls, adjusting for age and education. Values to the left of 1.0 indicate decreased odds of belonging to a clinical group per unit increase in fluency score. In the forest plot, filled circles denote statistically significant effects (*p* <.05), whereas open circles represent non-significant effects

For every one‐unit increase in semantic fluency, the odds of belonging to a clinical group decreased by 9.3% for SCD, 24.3% for MCI, and 32.9% for dementia relative to healthy controls (Table 4). Similarly, higher discrepancy scores reduced the odds of being in Group 1 (SCD) by 7.7%, Group 2 (MCI) by 19%, and Group 3 (Dementia) by 24%. In contrast, phonemic fluency scores significantly lowered the odds for MCI (14.5% decrease) and dementia (27.5% decrease) but did not significantly affect the odds of SCD. These findings underscore that, while semantic fluency and discrepancy scores reliably differentiate SCD from healthy controls, phonemic fluency appears less sensitive to early cognitive changes associated with SCD (Fig. 2).

**Table 4:** Multinomial logistic regression results for diagnostic group membership predictors

Predictor/Measure	Comparison Group	Percent Decrease	Relative Risk Ratio (RRR)	95% Confidence Interval	p-value
Semantic Fluency	SCD vs. Healthy Controls	9.3% decrease	0.907	[0.843, 0.976]	0.009
Semantic Fluency	MCI vs. Healthy Controls	24.3% decrease	0.757	[0.668, 0.857]	< 0.001
Semantic Fluency	Dementia vs. Healthy Controls	32.9% decrease	0.671	[0.591, 0.762]	< 0.001
VF Discrepancy	SCD vs. Healthy Controls	7.7% decrease	0.923	[0.858, 0.994]	0.034
VF Discrepancy	MCI vs. Healthy Controls	19.0% decrease	0.810	[0.704, 0.932]	0.003
VF Discrepancy	Dementia vs. Healthy Controls	24.0% decrease	0.760	[0.673, 0.860]	< 0.001
Phonemic Fluency	SCD vs. Healthy Controls	No significant difference	0.976	[0.908, 1.050]	0.501
Phonemic Fluency	MCI vs. Healthy Controls	14.5% decrease	0.855	[0.748, 0.978]	0.022
Phonemic Fluency	Dementia vs. Healthy Controls	27.5% decrease	0.725	[0.635, 0.827]	< 0.001

*SCD* Subjective Cognitive Decline, *MCI* Mild Cognitive Impairment, *VF* Verbal Fluency, *RRR* Relative Risk Ratio, *CI* Confidence Interval. Percent decrease indicates the decrease in odds per unit increase in fluency measure. All analyses controlled for age and education. P-values are reported to three decimal places unless p <.001

### Diagnostic classification analyses: confusion matrix

The confusion matrix gives the raw, detailed picture of errors by class (who is confused with whom). We then compute condensed summaries—Recall, Precision, F1, and Balanced Accuracy—to quantify how big those errors are for each class and overall. These answer complementary questions: the matrix shows where the errors are; the metrics summarize their magnitude.

Based on a confusion matrix derived from the multinomial logistic regression model, diagnostic performance varied according to the fluency measure. The SCD group was best identified using the VF discrepancy score (49.3% accuracy), while healthy controls were most accurately classified via phonemic fluency (63.9%), and dementia through semantic fluency (78.9%). In contrast, MCI was not successfully classified by any measure (0% accuracy). Across all metrics, the most frequent misclassifications occurred between healthy controls and SCD, with MCI predominantly misclassified as dementia. Moreover, semantic fluency often confused dementia with SCD, whereas both VF discrepancy and phonemic fluency tended to misclassify dementia as healthy controls. These results highlight that while each fluency measure has its unique diagnostic strengths, VF discrepancy appears particularly useful for detecting SCD.

### Diagnostic classification analyses: classification performance metrics

To evaluate performance in our multi-class setting, we calculated balanced accuracy, F1, recall, and specificity using a one-vs-all approach. In this framework, each diagnostic category was treated as the positive class while all remaining classes were grouped as negative.

As shown in Table 5, for individuals with SCD, the Verbal Fluency discrepancy measure performed best, showing a balanced accuracy of 0.609, an F1-score of 0.520, recall of 0.493, and specificity of 0.724. In contrast, all measures had difficulty detecting MCI (all had zero recall), indicating that none were able to correctly identify those with mild impairment. For dementia, semantic fluency stood out with a balanced accuracy of 0.810, recall of 0.789, and specificity of 0.830, indicating it excels at identifying and correctly ruling out more advanced impairment. Lastly, healthy controls were reasonably well-classified across fluency measures, but again the discrepancy measure achieved the highest balanced accuracy (0.607), underlining its overall effectiveness at distinguishing normal function from decline.

**Table 5:** Classification performance metrics for fluency measures across diagnostic groups

Metric	Fluency Type	HC	SCD	MCI	Dementia
Balanced Accuracy	Semantic	0.602	0.559	0.500	0.810
	Phonemic	0.599	0.583	0.500	0.754
	VF Discr	0.607	0.609	0.500	0.736
F1	Semantic	0.558	0.451	0.000	0.723
	Phonemic	0.568	0.467	0.000	0.636
	VF Discr	0.570	0.520	0.000	0.612
Recall	Semantic	0.597	0.448	0.000	0.789
	Phonemic	0.639	0.418	0.000	0.737
	VF Discr	0.625	0.493	0.000	0.684
Specificity	Semantic	0.606	0.670	1.000	0.830
	Phonemic	0.560	0.747	1.000	0.771
	VF Discr	0.590	0.724	1.000	0.788

VF Discr. = Verbal Fluency Discrepancy. Performance metrics include balanced accuracy, F1-score, recall, and specificity values, all of which range from 0 to 1, where higher values indicate better performance. Metrics were computed for each diagnostic category using a one-vs-all approach. Recall (sensitivity): the proportion of true cases correctly identified. Specificity: the proportion of non-cases correctly ruled out. Precision: of all predicted “case” labels, the proportion that are truly cases. F1-score: a single number that combines precision and recall and is high only when both are high. Balanced accuracy: the average of sensitivity and specificity per class in our one-vs-all setup, giving equal weight to cases and non-cases in imbalanced data. Because group sizes were unequal, overall accuracy can appear better simply by getting the common class right; balanced accuracy avoids that bias

*HC* Healthy Controls, *SCD* Subjective Cognitive Decline, *MCI* Mild Cognitive Impairment

### Diagnostic classification analyses: precision-recall curve

Using a (One vs. all) Precision-Recall Curve analysis derived from our multinomial logistic regression model, we assessed the discriminative power of three fluency measures across diagnostic groups (see Table 6). The precision-recall curve (PRC) is better suited for highlighting a model’s ability to identify the minority class accurately in imbalanced datasets.

**Table 6:** Precision Recall Area Under the Curve (AUC) for Verbal Fluency Measures Across Diagnostic Groups

Group	Semantic Fluency	Phonemic Fluency	VF Discrepancy	PRC Baseline Score*
Healthy Control	0.629	0.573	0.583	0.375
SCD	0.534	0.488	0.571	0.347
MCI	0.231	0.179	0.183	0.0833
Dementia	0.756	0.701	0.619	0.198

Higher AUC values indicate better discrimination between groups. * The score that a random classifier would achieve (e.g. a classifier that does not consider any underlying patterns in the input data)

*AUC* Area Under the Curve, *VF* Verbal Fluency, *SCD* Subjective Cognitive Decline, *MCI* Mild Cognitive Impairment

**Fig. 3:**
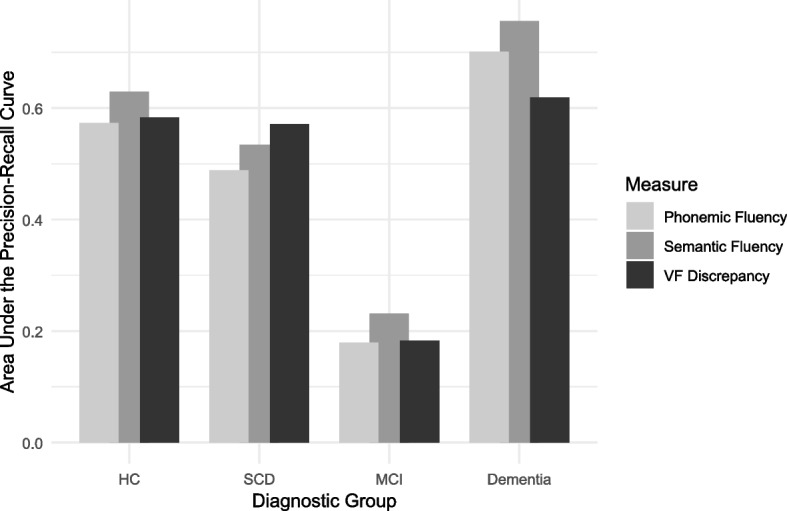
Precision–recall AUC values for each fluency measure across diagnostic groups. Note. Bars show area under the precision–recall curve (AUC‑PR) for predicting each diagnostic category (HC = healthy controls; SCD = subjective cognitive decline; MCI = mild cognitive impairment; Dementia) using semantic fluency, phonemic fluency, and the verbal‑fluency discrepancy score. Higher AUC‑PR indicates better discrimination performance. Grey shades correspond to the legend inside the graphic

Importantly, for the SCD group, VF discrepancy scores yielded the highest AUC-PR (0.571), outperforming both semantic and phonemic fluency in identifying these individuals. In comparison, semantic fluency was most effective for healthy controls with an AUC-PR of 0.629 and was the strongest predictor for dementia with an AUC-PR of 0.756. Notably, MCI (Group 2) was poorly classified by all measures, achieving a maximum AUC-PR of only 0.231 with semantic fluency, (one reason for that could be the low number of individuals in that group). Overall, these results underscore that while semantic fluency excels at distinguishing the extremes of cognitive function, VF discrepancy stands out as the most sensitive VF measure for identifying SCD (Fig. 3). In contrast to ROC curves, which have a constant baseline, the baseline value (y) in PRC plots is defined as the proportion of positive examples relative to the sum of positive (P) and negative (N) examples, calculated as y = P/(P + N) [23]. For SCD, the baseline was 0.347 and VF discrepancy achieved PR-AUC = 0.571, indicating modest above-baseline discrimination rather than strong clinical utility.

## Discussion

While many studies have examined semantic and phonemic fluency in MCI and dementia, fewer have explored whether these measures can reveal subtle deficits in SCD, particularly regarding the utility of discrepancy scores in clinical settings. Our study addressed this gap by comparing semantic and phonemic fluency between healthy controls and individuals with SCD, MCI, or dementia. We further evaluated whether these measures—and their difference—could reliably distinguish SCD from the other groups. Consistent with our first hypothesis, healthy controls demonstrated significantly better semantic fluency than SCD participants, whereas phonemic fluency did not differ between groups. Both fluency types were reduced in MCI and dementia, with semantic declines being especially pronounced. MCI proved the most difficult to differentiate (likely due to a smaller sample size), whereas semantic fluency scores were the most accurate in classifying dementia. Notably, the VF discrepancy score was the most effective single measure for detecting SCD, although its diagnostic accuracy was modest above-baseline discrimination. These findings indicate that VF discrepancy provided the highest PR-AUC and balanced accuracy for SCD. Given their modest incremental discrimination, we make no clinical utility claims and recommend external validation. There is consensus that semantic fluency is the most sensitive fluency marker in dementia [9] [24]. Both semantic fluency and the VF discrepancy score also hold key diagnostic value in MCI [25, 26, 11]. Our most novel findings relate to SCD, so the remainder of this discussion will focus on SCD‐related findings.

Several studies support our results: compared to healthy controls, SCD individuals consistently show lower baseline semantic fluency (usually assessed by animal naming), whereas phonemic fluency remains relatively spared [27, 28, 29, 30] [31, 32, 33, 34, 35]. These studies often recruited participants who met one or more SCD-plus criteria (e.g., later onset, stability, worry, or memory-clinic referral), further supporting a heightened risk of subsequent decline [36]. Although animal naming is considered a sensitive semantic category, research suggests that the choice of category is essential [37]. For example, Macoir et al. [30] found significant group differences using a famous faces naming task, whereas De Simone et al. [38], using a composite score from fruits, colors, and animals, observed only trend-level differences in semantic fluency and significant differences in the verbal fluency discrepancy score.

Other investigations have yielded contradictory results. Nikolai et al. [27] observed baseline differences only in vegetable-naming scores — and not in animal fluency — in their clinical SCD group, which met most of Jessen’s SCD-plus criteria. In contrast to our approach, which involved recruiting control participants without cognitive complaints from the same clinical setting and utilizing minimally adjusted raw fluency scores, their study recruited from a separate normative aging study and applied stricter exclusion criteria and score transformations. These methodological differences may have had subtle effects. Moreover, Nikolai et al. found negligible group differences on the Geriatric Depression Scale, whereas our SCD cohort showed substantially higher depression and anxiety, which may have further influenced fluency performance. Additionally, some studies with small samples [39, 40, 41], or who combined multiple semantic categories [39] or fluency types (e.g., semantic and phonemic) [40] into a single measure, or low adherence to SCD-plus criteria [40, 41] found no significant differences in semantic fluency. In one study, participants with SCD outperformed healthy controls, likely because they had significantly more years of education [42]. One extensive sample clinical study found no significant difference in animal naming between healthy controls and individuals with subjective memory complaints [43]. One explanation is that they did not strictly follow Jessen’s guidelines for defining SCD. Another is that their SCD group had lower educational attainment (mean ∼9 years), compared to our predominantly highly educated sample. This distinction is crucial, as a meta-analysis [44] has shown that individuals with SCD who have at least 15 years of education are at an increased risk of objective cognitive decline, as they are more aware of their cognitive decline.

Regarding our second hypothesis, few studies have assessed the discriminative power of VF discrepancy scores in SCD. López-Higes et al. [45] is the only study to report on this question, finding an ROC AUC of 0.572 for VF discrepancy scores in distinguishing healthy controls from SCD individuals—a value considered a failure (< 0.60) by standard ROC guidelines [46]. In contrast to the prior study, we used a clinical sample and a precision–recall approach, which is more sensitive to both minority classes and group imbalances. We produced a VF discrepancy AUC of 0.571. It is important to emphasize that PR-AUC is prevalence-dependent and lacks standardized cutoffs, so we interpret it relative to class prevalence rather than ROC AUC rules of thumb. With SCD representing about 34.7% of participants, a random classifier would have a baseline precision of 0.347 [23]. In our data, the VF discrepancy PR-AUC = 0.571 indicates modest above-baseline discrimination, not “poor” performance under an AUROC ≥ 0.60 rule. However, this level of discrimination does not support strong standalone clinical utility. Its overall discriminative power remains moderate, with a balanced accuracy of 0.609. However, this is the highest score across all fluency measures, outperforming predictions based solely on semantic fluency scores. Future studies could benefit from harmonizing SCD operational definitions, carefully selecting semantic categories, and comparing raw versus standardized VF metrics. This would better clarify the precise role of semantic fluency—and the VF discrepancy score—in identifying incipient cognitive decline.

Beyond clinical applications, our results align with broader evidence that semantic memory measures are particularly sensitive to early cognitive decline in SCD. Although the link between amyloid accumulation and semantic fluency is modest [47], studies show that semantic cognition is disproportionately affected by amyloid and can reveal subtle deficits missed by other tests [48, 49, 50, 51]. Because semantic memory is typically preserved in normal aging, even minor declines may signal pathological change [52, 53]. This vulnerability likely reflects early neurofibrillary tangle formation in AD or Primary age-related tauopathy in regions critical for semantic processing, such as the perirhinal cortex [54, 55]. Aligned with the vital role of semantic memory in early cognitive decline, our findings show that semantic fluency — and, in particular, the VF discrepancy score — was the most sensitive VF measure in detecting individuals with SCD.

## Conclusion

Our study reveals that individuals with SCD exhibit objective deficits in semantic fluency, but not in phonemic fluency, and that VF discrepancy scores are more predictive of SCD than semantic fluency scores alone. Given their moderate accuracy, VF discrepancy scores are not clinically useful as standalone tests; external validation is needed to determine whether incorporating them into multi-test batteries improves detection of objective cognitive decline in SCD. This is important because objective cognitive deficits may serve as an early indicator of future MCI or dementia in individuals with SCD. Given the rapidly growing prevalence of dementia worldwide, incorporating a discrepancy-based verbal fluency measure into standard screenings could offer a cost-effective way to identify individuals who may benefit most from early interventions. Future studies should combine the VF discrepancy measure with complementary neuropsychological tests (e.g., memory recall and executive function) and standardize semantic category selection to evaluate whether diagnostic precision and clinical utility improve. This multimodal approach is likely to yield more robust cognitive batteries for the early detection of objective cognitive decline.

## Limitations

A clear strength of this investigation is the inclusion of four diagnostically distinct groups drawn from the same memory-clinic pathway, which standardized testing conditions; nonetheless, several limitations temper the conclusions. Power analyses revealed that the study was underpowered (see Methods section), due to modest sample sizes across most groups, especially the MCI, SCD, and control groups, suggesting that future work should recruit more participants per group. Interpretation is further limited by unmatched age and education profiles (adjusted for statistically but still a potential source of residual confounding) and by the fact that our cohort was predominantly highly educated, which restricts the generalizability of our findings to broader populations. Because no CSF or PET biomarkers were collected in the SCD and control populations, underlying neuropathology could not be confirmed, and the elevated depression and anxiety scores observed in the SCD group may have attenuated fluency performance, potentially inflating the apparent SCD effect. Finally, the cross-sectional design prevents causal inference or direct prediction of progression; longitudinal studies with biomarker integration and larger, demographically balanced samples will therefore be essential to clarify trajectories from SCD to MCI or dementia and to validate verbal-fluency metrics as early detection tools.

## Data Availability

The raw dataset is available upon reasonable request sent to the corresponding author.

## Abbreviations

ACE: Addenbrooke’s Cognitive Examination
AD: Alzheimer’s disease
ADL: Activities of daily living
ANCOVA: Analysis of covariance
AUC-PR: Area under the precision–recall curve
BDI: Beck Depression Inventory
CI: Confidence interval
CSF: Cerebrospinal fluid
HC: Healthy controls
IADL: Instrumental activities of daily living
MCI: Mild cognitive impairment
MMSE: Mini-Mental State Examination
MRI: Magnetic resonance imaging
PET: Positron-emission tomography
PF: Phonemic fluency
PRC: Precision–recall curve
RAVLT: Rey Auditory Verbal Learning Test
RRR: Relative risk ratio
SCD: Subjective cognitive decline
SD: Standard deviation
SF: Semantic fluency
STAI: Spielberger State–Trait Anxiety Inventory
STAI-S: State Anxiety subscale of STAI
STAI-T: Trait Anxiety subscale of STAI
VF: Verbal fluency
VFD: Verbal-fluency discrepancy score (semantic − phonemic)
VLOM ratio: Verbal fluency + Language / Orientation + Memory ratio (ACE composite)

## References

[1] Petersen RC, Lopez O, Armstrong MJ, Getchius TSD, Ganguli M, Gloss D, et al. Practice guideline update summary: mild cognitive impairment. Neurology. 2018;90(3):126–35. 10.1212/WNL.0000000000004826. 29282327 10.1212/WNL.0000000000004826 PMC5772157

[2] Pike KE, Cavuoto MG, Li L, Wright BJ, Kinsella GJ. Subjective cognitive decline: level of risk for future dementia and mild cognitive impairment, a meta-analysis of longitudinal studies. Neuropsychol Rev. 2022;32(4):703–35. 10.1007/s11065-021-09522-3. 34748154 10.1007/s11065-021-09522-3

[3] Gustavsson A, Norton N, Fast T, Frölich L, Georges J, Holzapfel D, et al. Global estimates on the number of persons across the Alzheimer’s disease continuum. Alzheimers Dement. 2023;19(4):658–70. 10.1002/alz.12694. 35652476 10.1002/alz.12694

[4] Livingston G, Huntley J, Liu KY, Costafreda SG, Selbæk G, Alladi S, et al. Dementia prevention, intervention, and care: 2024 report of the Lancet standing commission. Lancet. 2024;404(3):572–628. 10.1016/S0140-6736(24)01296-0. 39096926 10.1016/S0140-6736(24)01296-0

[5] Rafii MS, Aisen PS. Detection and treatment of Alzheimer’s disease in its preclinical stage. Nat Aging. 2023;3(5):520–31. 10.1038/s43587-023-00410-4. 37202518 10.1038/s43587-023-00410-4 PMC11110912

[6] Molinuevo JL, Rabin LA, Amariglio R, Buckley R, Dubois B, Ellis KA, et al. Implementation of subjective cognitive decline criteria in research studies. Alzheimers Dement. 2017;13(3):296–311. 10.1016/j.jalz.2016.09.012. 27825022 10.1016/j.jalz.2016.09.012 PMC5344703

[7] Stark M, Wolfsgruber S, Kleineidam L, Frommann I, Altenstein S, Bartels C, et al. Relevance of minor neuropsychological deficits in patients with subjective cognitive decline. Neurology. 2023;101(17):e2185–96. 10.1212/WNL.0000000000207844. 37821235 10.1212/WNL.0000000000207844 PMC10663030

[8] Schmidt CSM, Schumacher LV, Römer P, Leonhart R, Beume L, Martin M, et al. Are semantic and phonological fluency based on the same or distinct sets of cognitive processes? Insights from factor analyses in healthy adults and stroke patients. Neuropsychologia. 2017;99:148–55. 10.1016/j.neuropsychologia.2017.02.019. 28257876 10.1016/j.neuropsychologia.2017.02.019

[9] Henry JD, Crawford JR, Phillips LH. Verbal fluency performance in dementia of the Alzheimer’s type: a meta-analysis. Neuropsychologia. 2004;42(9):1212–22. 10.1016/j.neuropsychologia.2004.02.001. 15178173 10.1016/j.neuropsychologia.2004.02.001

[10] Vaughan RM, Coen RF, Kenny R, Lawlor BA. Preservation of the semantic verbal fluency advantage in a large population-based sample: normative data from the TILDA study. J Int Neuropsychol Soc. 2016;22(6):570–6. 10.1017/S1355617716000291. 27055803 10.1017/S1355617716000291

[11] Wright LM, De Marco M, Venneri A. Verbal fluency discrepancies as a marker of the prehippocampal stages of Alzheimer’s disease. Neuropsychology. 2023;37(6):790–800. 10.1037/neu0000836. 35737533 10.1037/neu0000836

[12] Jessen F, Amariglio RE, van Boxtel M, Breteler M, Ceccaldi M, Chételat G, et al. A conceptual framework for research on subjective cognitive decline in preclinical Alzheimer’s disease. Alzheimers Dement. 2014;10(3):844–52. 10.1016/j.jalz.2014.01.001. 24798886 10.1016/j.jalz.2014.01.001 PMC4317324

[13] Jessen F, Amariglio RE, Buckley RF, van der Flier WM, Han Y, Molinuevo JL, et al. Characterisation of subjective cognitive decline. Lancet Neurol. 2020;19(3):271–8. 10.1016/S1474-4422(19)30368-0. 31958406 10.1016/S1474-4422(19)30368-0 PMC7062546

[14] Petersen RC, Roberts RO, Knopman DS, Boeve BF, Geda YE, Ivnik RJ, et al. Mild cognitive impairment: ten years later. Arch Neurol. 2009;66(12):1447–55. 10.1001/archneurol.2009.266. 20008648 10.1001/archneurol.2009.266 PMC3081688

[15] Kaszás B, Fekete J. Validation of the Hungarian version of Addenbrooke’s Cognitive Examination for detecting major and mild neurocognitive disorders. Int Neuropsychiatr Dis J. 2020;14(4):79–88. Available from: https://journalindj.com/index.php/INDJ/article/view/277. Cited 2025 Apr 20

[16] Creavin ST, Wisniewski S, Noel-Storr AH, Trevelyan CM, Hampton T, Rayment D, et al. Mini-mental state examination (MMSE) for the detection of dementia in clinically unevaluated people aged 65 and over in community and primary care populations. Cochrane Database Syst Rev. 2016;2016:CD011145.pub2. 10.1002/14651858.CD011145.pub2. 26760674 10.1002/14651858.CD011145.pub2 PMC8812342

[17] Strauss E, Sherman EMS, Spreen O. A compendium of neuropsychological tests: administration, norms, and commentary. 3rd ed. New York: Oxford University Press; 2006. xvii, 1216 pp.

[18] Berente DB, Zsuffa J, Werber T, Kiss M, Drotos A, Kamondi A, et al. Alteration of visuospatial system as an early marker of cognitive decline: a double-center neuroimaging study. Front Aging Neurosci. 2022;14:854368. 10.3389/fnagi.2022.854368. 35754966 10.3389/fnagi.2022.854368 PMC9226394

[19] Kónya A, Verseghi A, Rey T. Rey‑Emlékezeti Vizsgálatok. Budapest: Pszicho‑Teszt Szerviz; 1995.

[20] Alladi S, Arnold R, Mitchell J, Nestor PJ, Hodges JR. Mild cognitive impairment: applicability of research criteria in a memory clinic and characterization of cognitive profile. Psychol Med. 2006;36(4):507–15. 10.1017/S0033291705006744. 16426486 10.1017/S0033291705006744

[21] Miklósi M, Martos T, Kocsis-Bogár K, Perczel-Forintos D. Psychometric properties of the Hungarian version of the Cognitive Emotion Regulation Questionnaire. Psychiatr Hung. 2011;26(2):102–11. 21653995

[22] Sipos K, Sipos M. The development and validation of the Hungarian form of the State-Trait Anxiety Inventory. In: Spielberger CD, Diaz-Guerrero R, editors. Cross-Cultural Anxiety, vol. 2. Washington (DC): Hemisphere Publishing Corp; 1983. p. 27–39.

[23] Saito T, Rehmsmeier M. The precision-recall plot is more informative than the ROC plot when evaluating binary classifiers on imbalanced datasets. PLoS ONE. 2015;10(3):e0118432. 10.1371/journal.pone.0118432. 25738806 10.1371/journal.pone.0118432 PMC4349800

[24] Sutin AR, Stephan Y, Terracciano A. Verbal fluency and risk of dementia. Int J Geriatr Psychiatry. 2019;34(7):863–7. 10.1002/gps.5081. 30729575 10.1002/gps.5081 PMC6530594

[25] Vaughan RM, Coen RF, Kenny R, Lawlor BA. Semantic and phonemic verbal fluency discrepancy in mild cognitive impairment: potential predictor of progression to Alzheimer’s disease. J Am Geriatr Soc. 2018;66(5):755–9. 10.1111/jgs.15294. 29572820 10.1111/jgs.15294

[26] McDonnell M, Dill L, Panos S, Amano S, Brown W, Giurgius S, et al. Verbal fluency as a screening tool for mild cognitive impairment. Int Psychogeriatr. 2020;32(8):1055–62. 10.1017/S1041610219000644. 31258101 10.1017/S1041610219000644 PMC9153280

[27] Nikolai T, Bezdicek O, Markova H, Stepankova H, Michalec J, Kopecek M, et al. Semantic verbal fluency impairment is detectable in patients with subjective cognitive decline. Appl Neuropsychol Adult. 2018;25(5):448–57. 10.1080/23279095.2017.1326047. 28548549 10.1080/23279095.2017.1326047

[28] Fagundo AB, López S, Romero M, Guarch J, Marcos T, Salamero M. Clustering and switching in semantic fluency: predictors of the development of Alzheimer’s disease. Int J Geriatr Psychiatry. 2008;23(10):1007–13. 10.1002/gps.2025. 18416452 10.1002/gps.2025

[29] Koppara A, Wagner M, Lange C, Ernst A, Wiese B, König H-H, et al. Cognitive performance before and after the onset of subjective cognitive decline in old age. Alzheimers Dement (N Y). 2015;1(3):194–205. 10.1016/j.dadm.2015.02.005. 10.1016/j.dadm.2015.02.005 PMC4876897 27239504

[30] Macoir J, Tremblay P, Beaudoin S, Parent M, Hudon C. Impaired lexical access for unique entities in individuals with subjective cognitive decline. Appl Neuropsychol Adult. 2024;25(5):1–11. 10.1080/23279095.2024.2344636. 10.1080/23279095.2024.2344636 38648449

[31] Maruta C, Martins IP. May subjective language complaints predict future language decline in community-dwelling subjects? Front Psychol. 2019;10:1974. 10.3389/fpsyg.2019.01974. 31555171 10.3389/fpsyg.2019.01974 PMC6722202

[32] Benito-León J, Mitchell AJ, Vega S, Bermejo-Pareja F. A population-based study of cognitive function in older people with subjective memory complaints. J Alzheimers Dis. 2010;22(1):159–70. 10.3233/JAD-2010-100972. 20847410 10.3233/JAD-2010-100972

[33] Açikgöz M, Özen Baru TB, Emre U, Taşçilar N, Atalay A, Köktürk F. Assessment of relation between subjective memory complaints and objective cognitive performance of elderly over 55 years of age. Noro Psikiyatr Ars. 2014;51(2):57–62. 10.4274/npa.y6719. 28360596 10.4274/npa.y6719 PMC5370255

[34] Kielb S, Rogalski E, Weintraub S, Rademaker A. Objective features of subjective cognitive decline in a United States national database. Alzheimers Dement. 2017;13(11):1337–44. 10.1016/j.jalz.2017.04.008. 28586648 10.1016/j.jalz.2017.04.008 PMC5712483

[35] Jutten RJ, Sikkes SAM, Amariglio RE, Buckley RF, Properzi MJ, Marshall GA, et al. Identifying sensitive measures of cognitive decline at different clinical stages of Alzheimer’s disease. J Int Neuropsychol Soc. 2021;27(4):426–38. 10.1017/S1355617720000934. 33046162 10.1017/S1355617720000934 PMC8041916

[36] Li H, Tan CC, Tan L, Xu W. Predictors of cognitive deterioration in subjective cognitive decline: evidence from longitudinal studies and implications for SCD-plus criteria. J Neurol Neurosurg Psychiatry. 2023;94(7):844–54. 10.1136/jnnp-2022-330246. 36868847 10.1136/jnnp-2022-330246

[37] Venneri A, Caroline J-C, Matteo DM, Davide Q, Marra C. Diagnostic and prognostic role of semantic processing in preclinical Alzheimer’s disease. Biomarkers Med. 2018;12(6):637–51. 10.2217/BMM-2017-0324. 10.2217/bmm-2017-0324 29896968

[38] De Simone MS, Rodini M, De Tollis M, Fadda L, Caltagirone C, Carlesimo GA. The diagnostic usefulness of experimental memory tasks for detecting subjective cognitive decline: preliminary results in an Italian sample. Neuropsychology. 2023;37(6):636–49. 10.1037/neu0000846. 35980693 10.1037/neu0000846

[39] Nutter-Upham KE, Saykin AJ, Rabin LA, Roth RM, Wishart HA, Pare N, et al. Verbal fluency performance in amnestic MCI and older adults with cognitive complaints. Arch Clin Neuropsychol. 2008;23(3):229–41. 10.1016/j.acn.2008.01.005. 18339515 10.1016/j.acn.2008.01.005 PMC2743541

[40] Rivera-Fernández C, Custodio N, Soto-Añari M. Neuropsychological profile in the preclinical stages of dementia: principal component analysis approach. Dement Neuropsychol. 2021;15(2):192–9. 10.1590/1980-57642021dn15-020006. 34345360 10.1590/1980-57642021dn15-020006 PMC8283881

[41] Atkins AS, Kraus MS, Welch M, Yuan Z, Stevens H, Welsh-Bohmer KA, et al. Remote self-administration of digital cognitive tests using the brief assessment of cognition: feasibility, reliability, and sensitivity to subjective cognitive decline. Front Psychiatry. 2022;13:910896. 10.3389/fpsyt.2022.910896. 36090378 10.3389/fpsyt.2022.910896 PMC9448897

[42] Linz N, Lundholm Fors K, Lindsay H, Eckerström M, Alexandersson J, Kokkinakis D. Temporal analysis of the Semantic Verbal Fluency Task in persons with subjective and mild cognitive impairment. In: Niederhoffer K, Hollingshead K, Resnik P, Resnik R, Loveys K, editors. Proceedings of the Sixth Workshop on Computational Linguistics and Clinical Psychology; 6 Jun 2019; Minneapolis, MN. Association for Computational Linguistics; 2019. p 103‑113. 10.18653/v1/W19-3012

[43] Park S, Lee J-H, Lee J, Cho Y, Park HG, Yoo Y, et al. Interactions between subjective memory complaint and objective cognitive deficit on memory performances. BMC Geriatr. 2019;19:1322. 10.1186/s12877-019-1322-9. 10.1186/s12877-019-1322-9 PMC6822458 31666029

[44] Wang XT, Wang ZT, Hu HY, Qu Y, Wang M, Shen XN, et al. Association of subjective cognitive decline with risk of cognitive impairment and dementia: a systematic review and meta-analysis of prospective longitudinal studies. J Prev Alzheimers Dis. 2021;8(2):277–85. 10.14283/jpad.2021.27. 34101784 10.14283/jpad.2021.27 PMC12280825

[45] López-Higes R, Rubio-Valdehita S, Fernandes SM, Rodrigues PFS. Differentiation between normal cognition and subjective cognitive decline in older adults using discrepancy scores derived from neuropsychological tests. Geriatrics. 2024;9(3):83. 10.3390/geriatrics9030083. 38920439 10.3390/geriatrics9030083 PMC11202516

[46] Nahm FS. Receiver operating characteristic curve: overview and practical use for clinicians. Korean J Anesthesiol. 2022;75(1):25–36. 10.4097/kja.21209. 35124947 10.4097/kja.21209 PMC8831439

[47] Vonk JMJ, Twait EL, Scholten R, Geerlings MI. Cross-sectional associations of amyloid burden with semantic cognition in older adults without dementia: a systematic review and meta-analysis. Mech Ageing Dev. 2020;192:111386. 10.1016/j.mad.2020.111386. 33091462 10.1016/j.mad.2020.111386 PMC7952036

[48] Baker JE, Lim YY, Pietrzak RH, Hassenstab J, Snyder PJ, Masters CL, et al. Cognitive impairment and decline in cognitively normal older adults with high amyloid-β: a meta-analysis. Alzheimers Dement (Amst). 2017;6(2):108–21. 10.1016/j.dadm.2016.09.002. 28239636 10.1016/j.dadm.2016.09.002 PMC5315443

[49] Papp KV, Rentz DM, Orlovsky I, Sperling RA, Mormino EC. Optimizing the preclinical Alzheimer’s cognitive composite with semantic processing: the PACC5. Alzheimers Dement (N Y). 2017;3(4):668–77. 10.1016/j.trci.2017.10.004. 29264389 10.1016/j.trci.2017.10.004 PMC5726754

[50] van den Berg RL, Butterbrod E, de Boer C, Schlüter LM, van Harten AC, Teunissen CE, et al. Amyloid-related changes in fluency in patients with subjective cognitive decline. Alzheimers Dement (Amst). 2025;17:e70063. 10.1002/dad2.70063. 39822289 10.1002/dad2.70063 PMC11736636

[51] Berg R, Butterbrod E, Boer C, Scheur D, Schlüter L-M, Harten A, et al. Amyloid-positivity is characterized by decline in semantic fluency: an in-depth investigation of verbal fluency trajectories, item-level characteristics and its prognostic value in patients with subjective cognitive decline. Alzheimers Dement. 2024;20(S3):e088963. 10.1002/alz.088963.

[52] Lövdén M, Rönnlund M, Wahlin A, Bäckman L, Nyberg L, Nilsson LG. The extent of stability and change in episodic and semantic memory in old age: demographic predictors of level and change. J Gerontol B Psychol Sci Soc Sci. 2004;59(3):P130–4. 10.1093/geronb/59.3.p130. 15118016 10.1093/geronb/59.3.p130

[53] Tromp D, Dufour A, Lithfous S, Pebayle T, Després O. Episodic memory in normal aging and Alzheimer disease: insights from imaging and behavioral studies. Ageing Res Rev. 2015;24:232–62. 10.1016/j.arr.2015.08.006. 26318058 10.1016/j.arr.2015.08.006

[54] Didic M, Barbeau EJ, Felician O, Tramoni E, Guedj E, Poncet M, et al. Which memory system is impaired first in Alzheimer’s disease? J Alzheimers Dis. 2011;27(1):11–22. 10.3233/JAD-2011-110557. 21799246 10.3233/JAD-2011-110557

[55] Jefferson-George KS, Wolk DA, Lee EB, McMillan CT. Cognitive decline associated with pathological burden in primary age-related tauopathy. Alzheimers Dement. 2017;13(8):1048–53. 10.1016/j.jalz.2017.01.028. 28322204 10.1016/j.jalz.2017.01.028 PMC5585025

